# Associations between body composition and gut microbiota in female college students with and without dance training

**DOI:** 10.1371/journal.pone.0350639

**Published:** 2026-06-02

**Authors:** Caifang Qiu, Hui Wang, Ran Liu

**Affiliations:** 1 College of Music, Shanghai Normal University, Shanghai, China; 2 Department of Pharmacology, School of Pharmacy, Nantong University, Nantong, China; 3 Department of Arts Education, Faculty of Education, East China Normal University, Shanghai, China; Mae Fah Luang University School of Anti Aging and Regenerative Medicine, THAILAND

## Abstract

Physical activity has been associated with gut microbiota variation and body composition phenotypes, but evidence in female dance students remains limited. This study compared body composition profiles and gut microbiota characteristics between female university students majoring in dance and those from non-dance majors. Seventy students were included (n = 35 per group). Body composition was assessed using bioelectrical impedance analysis (InBody 970), and fecal samples were analyzed by 16S rRNA gene sequencing. Dance students exhibited significantly lower adiposity related parameters and central fat accumulation indices, including PBF, BFM, FMI, VFA, VFL, WC, WHR, WHtR, ABSI, and conicity index (*P* < 0.001), while showing higher SMM/WT, TBW/WT, and lower limb lean mass distribution (*P* < 0.001). No significant differences were observed in FFM, SMM, or SMI (*P* > 0.05). Gut microbiota composition differed between groups, with differential taxa observed across multiple taxonomic levels. Notably, *Faecalibacterium* and *Lachnospiraceae_ND3007_group* showed negative correlations with adiposity related indices and positive correlations with muscle and hydration related parameters, whereas *Peptoniphilus*, *Ezakiella*, and *Fenollaria* were positively correlated with adiposity related indices. In addition, *Fusobacterium* and *Escherichia Shigella* were positively associated with central adiposity measures. These findings indicate that female dance students exhibit distinct body composition profiles, while microbiome-related differences and associations appear modest and exploratory, warranting further validation in larger and well-controlled studies.

## Introduction

The human gut microbiota plays an important role in host metabolism, nutrient processing, and immune regulation [1,2]. Advances in high-throughput sequencing technologies, particularly 16S rRNA gene sequencing, have substantially improved our understanding of the associations between gut microbial composition and host physiological characteristics [3]. Gut microbiota diversity and composition are influenced by multiple factors, including diet, age, geographic location, and lifestyle behaviors [4]. In addition, factors such as dietary patterns, supplement use, antibiotic exposure, and hormonal status have been reported to contribute to interindividual variability in gut microbiota composition, which may complicate the interpretation of group differences in observational studies [4–6].

Body composition is a key indicator of physical status and health, reflecting the relative distribution of fat mass, lean mass, and other tissue compartments [7]. In young adult women, body composition characteristics may vary considerably during the university years, and traditional anthropometric indices such as BMI may not fully capture these differences [8,9]. More detailed body composition parameters, including measures of adiposity, skeletal muscle distribution, and abdominal fat accumulation, provide a more comprehensive assessment of body composition phenotypes [10,11].

Physical activity has also been reported as a potential contributor to interindividual variation in gut microbiota composition, with several studies suggesting differences between physically active and sedentary populations [12–14]. Dance training represents a distinct form of structured physical activity that combines sustained practice, technical skill development, and repeated lower-limb–dominant movements. Previous studies have reported that individuals undergoing long-term dance training often exhibit distinct body composition characteristics compared with non-dance populations, including differences in body fat percentage and muscle-related indices [15,16]. However, while the relationship between physical activity and gut microbiota has been increasingly explored [12,14,17], evidence focusing specifically on dance students, particularly among young adult women, remains limited.

Therefore, this cross-sectional study aimed to examine the associations between dance training status, gut microbiota composition, and body composition in female university students. The primary objective was to compare gut microbiota community structure between dance and non-dance students. Secondary and exploratory objectives included identifying differentially abundant microbial taxa, examining their associations with detailed body composition parameters, and exploring predicted functional profiles. Gut microbiota composition was characterized using 16S rRNA gene sequencing, and body composition was assessed using bioelectrical impedance analysis (BIA), a non-invasive and widely used method in population-based studies that offers practical advantages in terms of feasibility and accessibility, although it may be influenced by hydration status compared with reference methods such as dual-energy X-ray absorptiometry (DXA).

## Materials and methods

### Participants

Participants consisted of 70 female college students, including 35 dance majors and 35 non-dance majors, all recruited from the same university during the same academic period. All participants were from the same academic year and were of similar age. The dancer group comprised students majoring in Chinese dance (classical and folk dance) who had received systematic and continuous dance training as part of their university curriculum. In contrast, the non-dancer group included students from other majors who had not received any formal dance training. None of the participants had a background in professional training for other sports. Aside from regular university physical education classes, participants did not engage in structured athletic training or consistent fitness programs. Participants were instructed to avoid antibiotic and probiotic use for at least 4 weeks prior to sample collection. Participants were required to have no changes in their dietary or lifestyle habits during the week prior to data collection. All participants were adults (≥ 18 years old), and no minors were included. This study was observational in design, and no intervention was applied.

This study was reviewed and approved by the Ethics Committee of Shanghai Normal University. All participants provided written informed consent prior to participation. Participation was voluntary, and students were informed that their decision to participate or not would not affect their grades or academic evaluation. All data were anonymized prior to analysis

### Body composition analysis

Body composition was assessed using a multi-frequency bioelectrical impedance analyzer (InBody 970, InBody Co., Ltd., Seoul, Republic of Korea) according to the manufacturer’s standard protocol. To minimize measurement variability, all assessments were conducted under standardized conditions. Participants were instructed to refrain from vigorous physical activity for at least 24 hours prior to testing. They were also asked to avoid food intake for at least 4 hours before measurement and to maintain a stable hydration status, avoiding excessive fluid intake. Participants were required to empty their bladder before testing. All measurements were performed at a consistent time of day to reduce the influence of diurnal variation. The following anthropometric and body composition parameters were obtained: body weight (kg), body mass index (BMI), percent body fat (PBF), body fat mass (BFM), fat mass index (FMI), fat-free mass (FFM), skeletal muscle mass (SMM), skeletal muscle index (SMI), and the ratio of skeletal muscle mass to body weight (SMM/WT). Indicators of abdominal adiposity and body shape included waist circumference (WC), waist-to-hip ratio (WHR), waist-to-height ratio (WHtR), visceral fat area (VFA), visceral fat level (VFL), abdominal body shape index (ABSI), and the conicity index. Segmental body composition parameters included the relative lean mass (%) of the right and left legs. In addition, parameters related to body water distribution and metabolism included total body water (TBW), the ratio of total body water to body weight (TBW/WT), intracellular water (ICW), extracellular water (ECW), protein, minerals, and basal metabolic rate (BMR).

### Sample collection

Fecal samples were collected from all participants using sterile collection kits. Participants were instructed on proper collection procedures and handed their samples to the study administrator immediately after collection. All samples were then stored at −80℃ by the study administrator until further processing.

### DNA extraction and 16S rRNA sequencing

Genomic DNA was extracted from fecal samples using the E.Z.N.A.® Soil DNA Kit (Omega Bio-Tek, USA) according to the manufacturer’s instructions. The V3–V4 hypervariable region of the 16S rRNA gene was amplified using universal primers 338F (5’-ACTCCTACGGGAGGCAGCAG-3’) and 806R (5’-GGACTACHVGGGTWTCTAAT-3’). PCR products were purified, quantified, and pooled in equimolar concentrations. Sequencing libraries were constructed using the NEXTFLEX Rapid DNA-Seq Kit (Bioo Scientific, USA) and sequenced on an Illumina NovaSeq 6000 platform using paired-end 150 bp reads.

### Bioinformatics analysis

Raw sequencing reads were quality-filtered using fastp (v0.20.0) and merged using FLASH (v1.2.7). Low-quality bases with a quality score below 20 were trimmed, and reads shorter than 50 bp or containing ambiguous bases were discarded. Paired-end reads were merged with a minimum overlap of 10 bp and a maximum mismatch ratio of 0.2. Processed sequences were analyzed using QIIME2 (v2024.10). Denoising, quality control, and chimera removal were performed using the DADA2 plugin to generate amplicon sequence variants (ASVs). Taxonomic classification was conducted using the SILVA 138 database with a naïve Bayes classifier. To account for differences in sequencing depth, data were rarefied to 18,000 reads per sample prior to downstream analyses. Alpha and beta diversity metrics were calculated using QIIME2, and PERMANOVA analysis was conducted using the adonis2 function in the vegan R package. Permutational analysis of multivariate dispersions (PERMDISP) was conducted using the betadisper function in the vegan R package to assess differences in within-group variability. Differential abundance analysis was performed using ANCOM-BC2 with false discovery rate (FDR) correction. LEfSe analysis (LDA > 2) was also conducted and interpreted as exploratory.

### Statistical analysis

Differences in body composition parameters between groups were assessed using t-tests or Mann–Whitney U tests. Correlation analysis between microbial taxa and body composition parameters was performed using Pearson correlation. P-values were adjusted for multiple comparisons using the FDR method. A *P <* 0.05 was considered statistically significant for all comparisons.

## Results

### Basic characteristics of the students

As shown in Table 1, a total of 70 female college students were included in the analysis, with 35 dance majors and 35 non-dance majors. There was no significant difference in age between the two groups (19.34 ± 0.83 vs. 19.60 ± 0.80 years, *P* = 0.1967). Dance majors were significantly taller than non-dance majors (168.75 ± 3.83 vs. 165.03 ± 6.04 cm, *P* = 0.0033). Although body weight tended to be lower in dance majors compared with non-dance majors (56.38 ± 5.03 vs. 60.04 ± 11.72 kg), this difference did not reach statistical significance (*P* = 0.0969). BMI was significantly lower in dance majors than in non-dance majors (19.81 ± 1.69 vs. 21.96 ± 3.66 kg/m^2^, *P* = 0.0029).

**Table 1:** Basic characteristics of female college students majoring in dance and non-dance (Mean±SD).

	Dancer(n = 35)	non-Dancer(n=35)	*P-*value
Age (year)	19.34 ± 0.83	19.60 ± 0.80	0.1967
Height (cm)	168.75 ± 3.83	165.03 ± 6.04	0.0033
Weight (kg)	56.38 ± 5.03	60.04 ± 11.72	0.0969
BMI (kg/m^2^)	19.81 ± 1.69	21.96 ± 3.66	0.0029

### Body composition profiles in dance and non-dance students

Body composition parameters assessed by bioelectrical impedance analysis are summarized in Table 2. Compared with non-dance students, dance students exhibited significantly lower overall adiposity, as indicated by PBF, BFM, and FMI (*P* < 0.001). Indicators of central fat accumulation also differed markedly between groups: VFA and VFL were significantly lower in dance students (*P* < 0.001). Consistently, WC, WHR, WHtR, ABSI, and conicity index were all significantly lower in dance students (*P* < 0.001).

**Table 2:** Body characteristic parameters in dancer and non dancer.

	Dance(n = 34^*^)	non-Dancer(n=35)	P value
PBF	26.16 ± 3.18	31.65 ± 4.87	< 0.0001
BFM	14.78 ± 2.53	19.37 ± 5.95	0.0002
FMI	5.20 ± 0.92	7.09 ± 2.09	0.0001
FFM	41.59 ± 3.53	40.68 ± 6.43	0.4751
SMM	22.54 ± 2.12	22.04 ± 3.84	0.5145
SMI	6.21 ± 0.44	6.07 ± 0.81	0.4099
VFA	59.46 ± 11.28	89.28 ± 32.85	< 0.0001
VFL	5.47 ± 1.24	8.49 ± 3.33	< 0.0001
WC	74.19 ± 3.64	81.84 ± 9.39	0.0001
WHR	0.82 ± 0.02	0.88 ± 0.05	< 0.0001
WHtR	0.44 ± 0.02	0.50 ± 0.05	< 0.0001
ABSI	0.08 ± 0.00	0.08 ± 0.00	< 0.0001
Conicity Index	1.18 ± 0.03	1.25 ± 0.05	< 0.0001
SMM/WT	40.00 ± 1.93	36.93 ± 2.50	< 0.0001
Lean Mass (%) Right Leg	108.55 ± 5.21	99.14 ± 7.91	< 0.0001
Lean Mass (%) Left Leg	108.31 ± 5.23	99.10 ± 7.82	< 0.0001
TBW/WT	53.98 ± 2.38	49.98 ± 3.67	< 0.0001
BMR	1268.26 ± 76.30	1248.71 ± 138.85	0.4765

*One dancer student InBody data was missing.

In contrast, no significant differences were observed in FFM, SMM, or SMI (*P* > 0.05). However, SMM/WT was significantly higher in dance students (*P* < 0.001). Segmental analysis showed that the relative lean mass of both the right and left legs was significantly greater in dance students (*P* < 0.001). Regarding body water distribution, TBW/WT was significantly higher in dance students (*P* < 0.001), whereas BMR did not differ significantly between groups (*P* = 0.4765).

### Gut microbiota diversity and overall community structure

**Fig 1:**
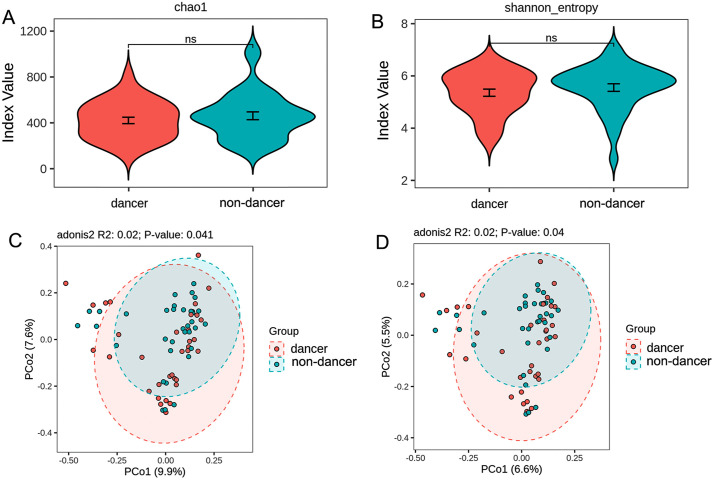
Alpha and beta diversity of gut microbiota in dance and non-dance students (A–B: Chao1 and Shannon indices; C–D: Bray–Curtis- and Jaccard-based PCoA).

Alpha diversity of the gut microbiota was assessed using the Chao1 and Shannon indices. No statistically significant differences were observed between dance and non-dance students for either index (*P* > 0.05; Fig 1A-B).

Beta diversity was evaluated using Bray–Curtis dissimilarity and Jaccard distance and visualized by principal coordinate analysis (PCoA). Based on Bray–Curtis dissimilarity, a modest but statistically significant difference in overall microbial community structure was observed between dance and non-dance students (PERMANOVA: R^2^ = 0.02, *P* = 0.041; Fig 1C). A similar pattern was observed using Jaccard distance (PERMANOVA: R^2^ = 0.02, *P* = 0.040; Fig 1D). However, dispersion analysis based on Bray–Curtis distance indicated a significant difference in within-group variability between the two groups suggesting that the observed beta diversity difference may be partially influenced by dispersion.

**Fig 2:**
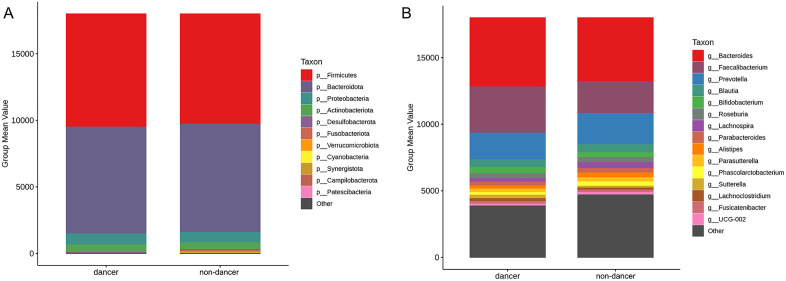
Bacterial composition in phylum(A) and genus (B) lever between dancers and non-dancers.

The overall composition of the gut microbiota at the phylum (A) and genus (B) levels is shown in Fig 2. At the phylum level, Firmicutes and Bacteroidota were the dominant taxa in both dance and non-dance students, together accounting for the majority of sequences, followed by Proteobacteria and Actinobacteriota. Other phyla, including Desulfobacterota, Fusobacteriota, and Verrucomicrobiota, were present at lower relative abundances.

At the genus level, *Bacteroides* was the most abundant genus in both groups, followed by *Faecalibacterium* and *Prevotella*. Notably, *Faecalibacterium* appeared to be more abundant in dance students compared with non-dance students, *Faecalibacterium* is generally considered a beneficial gut bacterium, particularly due to its role in butyrate production and its association with anti-inflammatory and metabolic health–related outcomes [18,19]. Other genera, including *Blautia*, *Bifidobacterium*, and *Roseburia*, were present at lower abundances. Overall, the gut microbiota of both groups showed similar taxonomic composition patterns at both the phylum and genus levels, with minor differences in the relative abundance of less dominant taxa.

### Differential gut microbial taxa between groups

**Fig 3:**
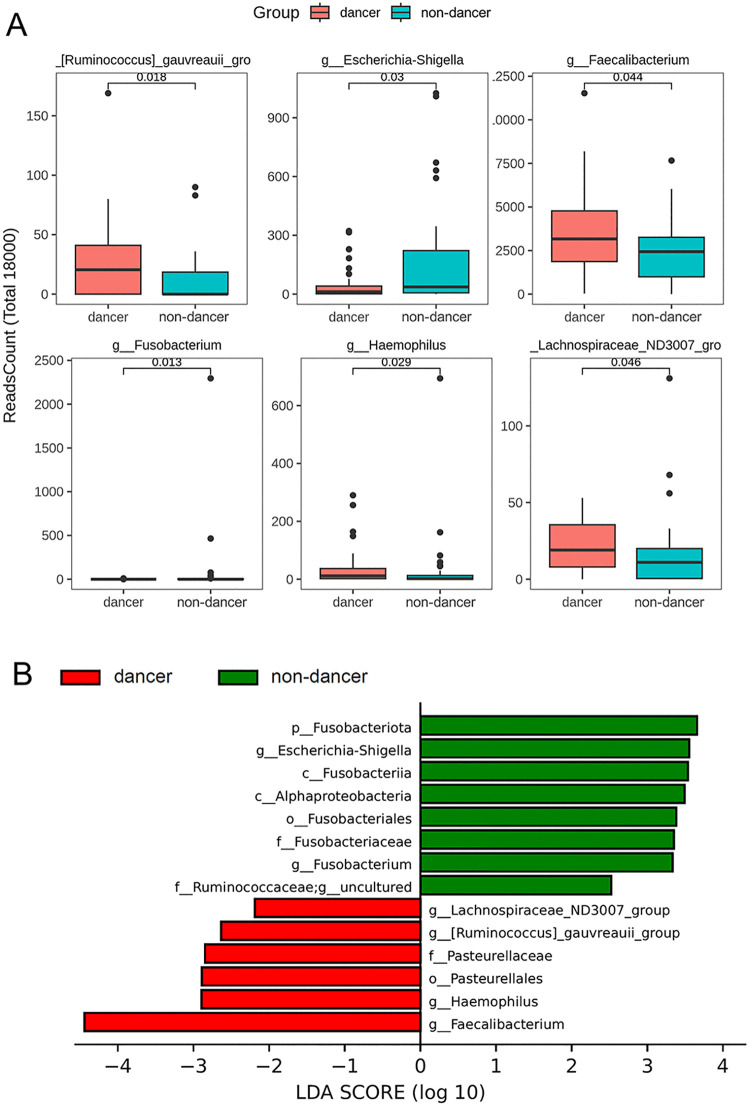
Differential gut microbial taxa between dance and non-dance students identified by genus-level comparison (A) and LEfSe analysis (B). Taxa with LDA scores > 2 were considered discriminative features.

Differential abundance of gut microbial taxa between dance and non-dance students was evaluated at the genus level and across taxonomic ranks. Genus-level comparisons are presented in Fig 3A, and LEfSe results are shown in Fig 3B. At the nominal significance level (*P* < 0.05), several genera showed differences in relative abundance between groups (Fig 3A). For example, *Faecalibacterium* appeared to be more abundant in dance students, whereas *Escherichia–Shigella* and *Fusobacterium* tended to be higher in non-dance students. Additional genera, including *Haemophilus*, *Lachnospiraceae ND3007* group, and the [Ruminococcus] gauvreauii group, also showed group differences at the nominal level. However, after adjustment for multiple comparisons using the FDR, none of these differences remained statistically significant, indicating that the overall compositional differences between groups were limited.

LEfSe analysis identified several taxa that distinguished the two groups at multiple taxonomic levels (Fig 3B). Taxa enriched in non-dance students were mainly related to the Fusobacteriota lineage, including *Fusobacteriota*, *Fusobacteriia*, *Fusobacteriales*, *Fusobacteriaceae*, and *Fusobacterium*, as well as *Escherichia–Shigella*. In contrast, taxa enriched in dance students included *Faecalibacterium*, *Haemophilus*, *Lachnospiraceae ND3007* group, the *[Ruminococcus] gauvreauii* group, and several related Firmicutes-associated taxa. Given the relatively small sample size and the susceptibility of LEfSe to false positives, these findings should be interpreted as exploratory rather than definitive.

### Predicted functional profiles of the gut microbiota

**Fig 4:**
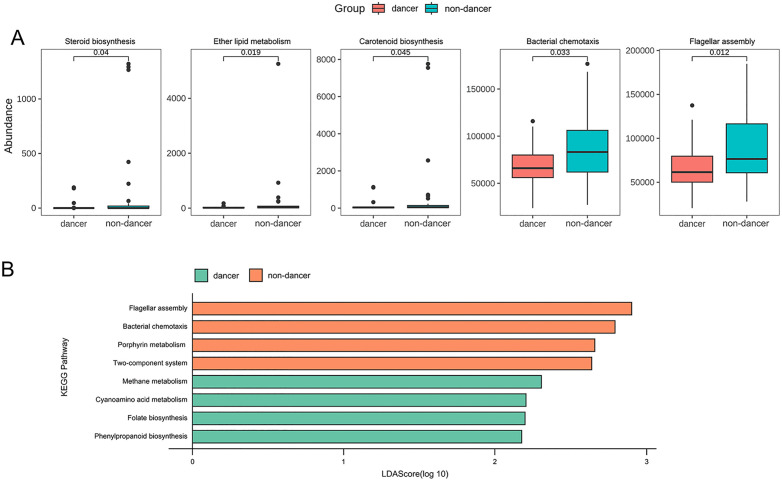
Predicted functional profiles of the gut microbiota in dance and non-dance students identified by PICRUSt2-based pathway comparison (A) and LEfSe analysis (B).

Predicted functional profiles of the gut microbiota were analyzed based on PICRUSt2 and are shown in Fig 4. Several KEGG pathways exhibited significant differences in predicted abundance between dance and non-dance students. As shown in Fig 4A, pathways related to steroid biosynthesis, ether lipid metabolism, and carotenoid biosynthesis differed significantly between groups (*P* < 0.05). In addition, pathways associated with bacterial chemotaxis and flagellar assembly also showed significant differences between dance and non-dance students (*P* < 0.05). However, these differences were based on uncorrected P-values and should be interpreted as exploratory.

LEfSe analysis further identified group-specific functional signatures (Fig 4B). Pathways such as flagellar assembly, bacterial chemotaxis, porphyrin metabolism, and the two-component system appeared to be more abundant in non-dance students, whereas pathways including methane metabolism, folate biosynthesis, and amino acid metabolism tended to be higher in dance students. Given the relatively small sample size and the susceptibility of LEfSe to false positives, these results should be considered exploratory.

### Associations between gut microbiota and body composition parameters

**Fig 5:**
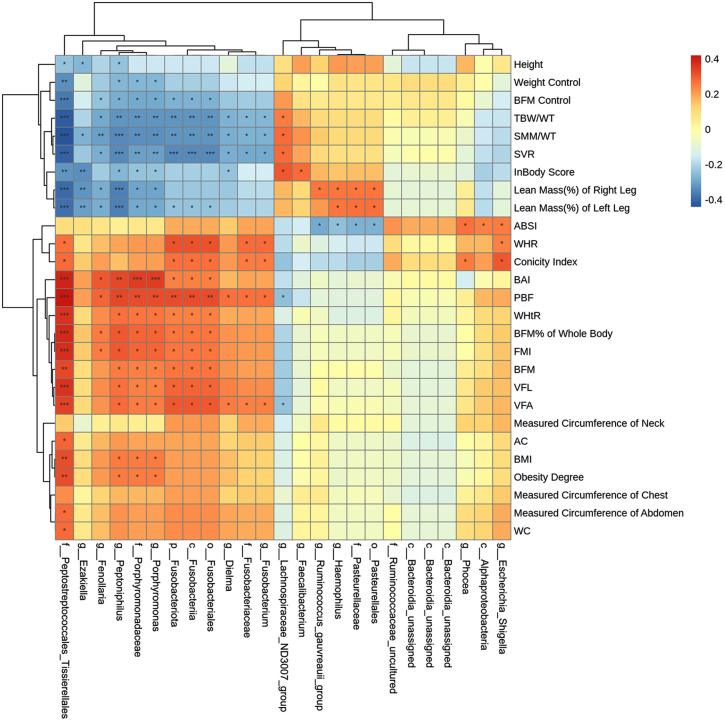
Correlations between differentially abundant gut microbial taxa and body composition parameters identified by Pearson correlation analysis. Correlation coefficients (Pearson’s r) are represented by the color scale.

Pearson correlation analysis was performed to explore associations between differentially abundant microbial taxa and body composition parameters that showed group differences. A total of 675 correlations were tested, and the results are presented as a clustered heatmap in Fig 5.

At the significance level (*P* < 0.05), several taxa showed associations with body composition parameters. In general, some taxa affiliated with Firmicutes appeared to be positively associated with adiposity-related indices, while taxa such as *Faecalibacterium* and *Lachnospiraceae_ND3007*_group tended to show inverse associations, being negatively related to adiposity measures and positively associated with indicators of lean mass and body water. Similar patterns were observed for several taxa within Bacteroidota and Proteobacteria, although the strength and direction of associations varied. While several associations were observed, these associations did not remain significant after FDR correction and should therefore be regarded as exploratory.

## Discussion

In this study, we compared body composition profiles and gut microbiota characteristics between female university students majoring in dance and those from non-dance majors. Dance students exhibited significantly lower adiposity related indices and central fat accumulation parameters, while showing higher relative skeletal muscle proportion and body water distribution indices. In parallel, group related differences in gut microbiota composition and predicted functional profiles were observed; however, these differences should be interpreted as associations rather than causal effects. Correlation analysis further suggested potential associations between differentially abundant microbial taxa and body composition parameters.

The observed differences in body composition are consistent with the training related phenotype of students undergoing long term structured physical activity. Notably, multiple adiposity related parameters such as PBF, FMI, VFA, and WC differed significantly between groups, whereas muscle mass indices such as SMM and SMI did not show significant differences. This pattern suggests that group differences may be driven primarily by fat mass and fat distribution rather than absolute skeletal muscle mass. Importantly, the use of detailed body composition parameters provided a more comprehensive characterization than BMI alone, supporting the value of multidimensional phenotyping in young adult women.

Exercise related microbiome research has reported that physical activity may be associated with gut microbiota features; however, findings across studies are heterogeneous. Recent human exercise studies consistently highlight that alpha diversity changes are not uniformly observed, while shifts in community composition and specific taxa are more frequently reported, and study level heterogeneity is substantial due to differences in populations, exercise prescription, dietary control, and analytical pipelines [20–22]. In the present study, overall taxonomic profiles at the phylum and genus levels appeared broadly similar between groups, whereas differential abundance analysis and predicted functional profiling suggested potential group related differences [21,22].

Beyond group comparisons, the correlation heatmap indicated patterns linking gut microbial taxa with adiposity related versus muscle or hydration related indices. Rather than isolated single taxon relationships, taxa tended to cluster according to correlation direction with central adiposity measures such as VFA, VFL, and WC versus indices reflecting relative muscle distribution and body water such as SMM/WT and TBW/WT. This pattern is in line with growing evidence that gut microbiota variation can be associated with skeletal muscle related phenotypes and body composition traits, including measures based on appendicular or relative muscle mass [23]. In our cohort, several Firmicutes affiliated taxa including g__Peptoniphilus, g__Fenollaria, g__Ezakiella, and f__Peptostreptococcales_Tissierellales showed positive correlations with adiposity related indices and negative correlations with SMM/WT, TBW/WT, and lower limb lean mass distribution, whereas g__Faecalibacterium, g__Lachnospiraceae_ND3007_group, and g__Ruminococcus_gauvreauii_group displayed the opposite pattern. Taxa within Bacteroidota, Fusobacteriota, and Proteobacteria also showed variable correlation patterns across body composition parameters.

Overall, female dance students showed distinct body composition profiles compared with non-dance students. Microbiome related differences were observed, but these findings should be interpreted with caution. In addition, BIA is sensitive to hydration status and physiological conditions, which may introduce measurement variability. Participants were not controlled for detailed dietary intake, supplement use, medication history, menstrual status, or precise training load. Therefore, observed microbiome differences may reflect broader lifestyle clustering rather than a specific effect of dance training itself. Finally, given the relatively small sample size and the number of comparisons performed, the possibility of false-positive findings cannot be excluded. Although multiple testing correction was applied, the absence of statistically robust associations suggests that these results should be interpreted as exploratory, the future work should use controlled diet assessment and multi-omics or longitudinal designs to test causality.

## Supporting information

S1 File Raw data. (XLSX)
